# *Treponema denticola* chymotrypsin-like protease as associated with HPV-negative oropharyngeal squamous cell carcinoma

**DOI:** 10.1038/s41416-018-0143-5

**Published:** 2018-06-22

**Authors:** Anna Kaisa Kylmä, Lauri Jouhi, Dyah Listyarifah, Hesham Mohamed, Antti Mäkitie, Satu Maria Remes, Caj Haglund, Timo Atula, Mikko T. Nieminen, Timo Sorsa, Jaana Hagström

**Affiliations:** 1 Department of Pathology, University of Helsinki, HUSLAB and Helsinki University Hospital, P.O. Box 21, 00014 Helsinki, Finland; 2 Department of Otorhinolaryngology—Head and Neck Surgery, University of Helsinki and Helsinki University Hospital, P.O. Box 263, 00029 Helsinki, Finland; 3 Department of Dental Biomedical Sciences, Faculty of Dentistry, Universitas Gadjah Mada, Jl. Denta Sekip Utara no 1, 55281 Yogyakarta, Indonesia; 4 Department of Medicine, Clinicum, University of Helsinki and Helsinki University Hospital, P.O. Box 63, 00014 Helsinki, Finland; 5 Department of Oral and Maxillofacial Diseases, University of Helsinki and Helsinki University Hospital, P.O. Box 41, 00014 Helsinki, Finland; 6 Division of Ear, Nose and Throat Diseases, Department of Clinical Sciences, Intervention and Technology, Karolinska Institutet and Karolinska Hospital, Stockholm, Sweden; 7 Department of Surgery, University of Helsinki and Helsinki University Hospital, P.O. Box 20, 00014 Helsinki, Finland; 8 Research Programs Unit, Translational Cancer Biology, University of Helsinki, P.O. Box 63, 00014 Helsinki, Finland; 9 Division of Periodontology, Department of Dental Medicine, Karolinska Institutet, Huddinge, Sweden

**Keywords:** Oral cancer, Tumour virus infections, Cancer microenvironment

## Abstract

**Background:**

An opportunistic oral pathogen, *Treponema denticola* (*Td*), has been linked to orodigestive carcinogenesis, but its role in oropharyngeal squamous cell carcinoma (OPSCC) has remained open. We evaluated the presence of *Td* chymotrypsin-like protease (*Td*-CTLP) in a series of 201 unselected consecutive OPSCC patients, and the relation of the *Td*-CTLP to human papillomavirus (HPV) status, to expression of toll-like receptors (TLR) 5, 7, and 9, and to clinical parameters and patient outcome.

**Methods:**

Clinicopathological data came from hospital registries. The expression of cell surface-bound *Td*-CTLP was evaluated by immunohistochemistry. Immunoexpression of TLRs 5, 7, and 9, and HPV status we studied earlier in this patient series.

**Results:**

We detected *Td*-CTLP in 81% of the OPSCC, and especially in HPV-negative tumours (48% of all OPSCCs). Among the HPV-positive tumours (52% of all OPSCCs), low *Td*-CTLP expression associated with low TLR 5 and high TLR 7 expression. Among those HPV-negative, higher TLR 5 and lower TLR 7 expression associated with high *Td*-CTLP expression. Strong *Td*-CTLP expression associated with poor disease-specific survival, but no similar association among HPV-positive and HPV-negative subgroups emerged.

**Conclusions:**

*Td*-CTLP was highly expressed in OPSCC and was associated with the HPV status of tumour tissue.

## INTRODUCTION

The incidence of oropharyngeal squamous cell carcinoma (OPSCC) associated with human papillomavirus (HPV) has increased,^1–6^ whereas the number of HPV-negative OPSCC cases has remained stable or has declined.^3,4^ The prognosis of patients carrying a HPV-positive tumour is better than the prognosis of patients with HPV-negative disease.^1,2^ Differences between these two OPSCC subgroups according to HPV have been studied widely, and in the latest WHO classification of head and neck tumours, these malignancies are classified as distinct entities.^7^ For the development of effective prevention and treatment strategies for both HPV-positive and HPV-negative OPSCC, a more detailed understanding of the cellular mechanisms and underlying causalities is essential.

Besides the known association of HPV with OPSCC, the role of several oral pathogens in oral and gastrointestinal tract cancers is evident. Epidemiological studies have shown an association between elevated periodontal pathogen *Porphyromonas gingivalis* serum IgG levels and increased risk of mortality from orodigestive cancer,^8^ as well as a correlation between elevated *P. gingivalis* plasma levels and increased incidence of pancreatic cancer.^9^ According to Narikiyo et al.,^10^ the oral pathogens *Streptococcus mitis*, *Streptococcus anginosus*, and *Treponema denticola* are frequent in oesophageal cancer tissue. Recently, *Td* has been evident also in oral and gastrointestinal tumour samples.^11^ Furthermore, *Fusobacterium nucleatum* has been associated with colorectal cancer and shown to promote colorectal carcinogenesis.^12^ Oral carcinogenesis, on the other hand, was promoted by *F. nucleatum* and *P. gingivalis* based on in vitro and in vivo studies via interaction with oral epithelial cells through toll-like receptors (TLR).^13^ The prevalence and role of oral pathogens in various OPSCC HPV subgroups calls for investigation.

During the last decades periodontitis has been associated with several systemic diseases, e.g., cardiovascular diseases,^14–17^ and with the development of diabetes mellitus type I and II complications.^18^ The mechanisms explaining the associations involve chronic low-grade systemic inflammation and oral microorganisms entering the bloodstream.^19–21^ *Td* is an invasive opportunistic pathogen belonging to a red complex group of oral pathogens identified in severe forms of periodontitis.^22^ Although *Td*, as well as other oral anaerobic spirochetes, may be present in the normal oral flora, its proportion increases significantly in periodontal infection.^22,23^ *Td* occurs in oesophageal cancer tissue^10^ and in squamous cell carcinomas (SCC) of the tongue, tonsils, and oesophagus, as well as in adenocarcinomas of the stomach, pancreas, and colon.^10,11^ *Td* possesses several virulence factors including an ability to adhere to epithelial cells and extracellular matrix components,^24–26^ to produce degrading enzymes,^27–30^ to secrete cytotoxic substances,^31^ to activate host-derived tissue-destructive matrix metalloproteinases,^11^ and to suppress local immune responses.^32^ One of its key virulence factors, a chymotrypsin-like protease (*Td*-CTLP), degrades a number of basement membrane components, thus enabling *Td* to invade epithelial tissue.^28^ The role of *Td* in the carcinogenesis of OPSCC is thus far unknown. However, the host’s systemic inflammatory response to chronic exposure to *Td* and its toxins,^33^ in combination with the proteolytic and immunomodulatory activities triggered by the bacteria, may be of significance.^11^

Our aim was to determine the prevalence of a cell surface-bound chymotrypsin-like proteinase (*Td*-CTLP), a key virulence factor of the oral pathogen *Td*, in 201 unselected consecutive OPSCC patients, and this factor’s relationship to HPV status, TLR 5, 7, and 9 expression, clinical parameters, and patient outcome.

## Materials and methods

### Patients and clinicopathological data

This patient cohort was previously described.^34^ To summarise, the cohort comprised 331 consecutive patients with oropharyngeal cancer diagnosed at the Helsinki University Hospital between 2000 and 2009. Inclusion of patients was restricted to the following ICD-10 codes: C01, C02.4, C05.1, C05.2, C05.8, C05.9, C09.0, C09.1, C09.8, C09.9, C10.0, C10.2, C10.3, C10.8, and C10.9. SCC and subtypes of SCC were included. Excluded from analysis were those patients with palliative intention of treatment (*n* = 44), concurrent head and neck squamous cell carcinoma (HNSCC) (*n* = 5), earlier treated HNSCC (*n* = 11), histology other than SCC (*n* = 18), or tumour tissue unavailability (*n* = 52).

Clinicopathological data from hospital registries, based on the earlier investigation on this same patient group, comprised patient age, sex, tumour histology, grade, UICC 7th edition TNM stage^35^; primary treatment, i.e., surgery (Sx), radiotherapy (RT), and chemotherapy (CRT); tumour recurrence, treatment of recurrent disease, and status at the last follow-up.^34^ Follow-up of all patients was at minimum 3 years or until death. Data on dates and causes of death came from Statistics Finland. The study received institutional permission and the approval of The Research Ethics Board at the Hospital District of Helsinki and Uusimaa.

In the group of 201 patients, 130 underwent primary surgery. Among these 130 patients 116 received also either RT or CRT as adjuvant oncological treatment. Five patients received no postoperative oncological therapy because of having Stage I–II disease and nine had none for case-related reasons. Definitive CRT or RT was delivered to 71 patients, and among them 11 underwent salvage surgery in the primary treatment phase (primary site, 1, neck only, 7, primary site and neck, 3). Tissue samples were collected before RT/CRT with the exception of two patients with only post-treatment samples available for immunohistochemistry.

HPV status and the expression of TLRs 5, 7, and 9 (TLR 5, TLR 7, TLR 9) had undergone analysis earlier by our research group.^34^ Briefly, HPV status was defined by Ventana Inform HPV in situ hybridisation (ISH) assay using a high-risk HPV probe and iVIEW Blue detection kit in Benchmark XT series stainer (Ventana Medical Systems, Inc., Tuscon, AZ, USA). This assay has affinity to these high-risk HPV subtypes: 16, 18, 31, 33, 35, 39, 45, 51, 56, 58, and 66. As a pretreatment, 5-μm thick sample sections were incubated with an extended Ventana cell-conditioning solution including ISH protease 3 (Ventana) for 32 min. HPV status was regarded as positive if any spot in ISH assay was positive. TLR status was studied by immunohistochemical staining by monoclonal mouse anti-human TLR 5 (1:200, IMG-664A, Imgenex, Port Coquitlam, BC, Canada), monoclonal rabbit anti-human TLR 7 (1:300, IMG-581A, Imgenex), and polyclonal rabbit anti-human TLR 9 (1:100, sc-25468, Santa Cruz Biotechnology, Inc., Dallas, TX, USA) as described.^14^

### Antibody preparation

*Td*-CTLP antibody was prepared according to the method of Grenier et al.^28^ The IgG fraction of the CTLP antibody was purified and washed on a Protein A-Sepharose^®^ CL-4B column (GE17-0780-01, Sigma-Aldrich Chemical Co LLC, St. Louis, MO, USA). The resulting anti-CTLP IgG fraction was tested for its specificity to *Treponema denticola* in immunofluorescence and ELISA assays with a closely related oral spirochete *Treponema vincentii* as a reference by a method described by Nieminen et al.^11^

### Immunohistochemistry for *Td*-CTLP

Preparation of the tissue microarray (TMA) blocks and immunohistochemical sample staining were performed as described by Nieminen et al.^11^ Polyclonal anti-CTLP IgG (1:1500 dilution) served as the antibody against *Td*-CTLP. Negative-control staining involved a non-immune species-specific Rabbit IgG (Vector Laboratories, Burlingame, CA, USA) and by omitting the primary antibody.

### Immunoscore

The decoded TMA blocks immunostained with *Td*-CTLP antibody were scored by two researchers (J.H. and A.K.K.) separately. Any discordance in scoring was solved by reassessment to achieve consensus. *Td*-CTLP scoring was based on intensity of the positivity in tumour tissue: none (0), mild (1), moderate (2), or strong (3). When several tumour spots were available for analysis, the choice was the highest positivity value for *Td*-CTLP. The positivity in *Td*-CTLP staining was localised in the cytoplasm of the carcinoma cells. In addition, immunopositivity was detectable in non-cancerous epithelial cells, secretory cells, and mononuclear inflammatory cells of some tissue samples, but these were not included in scoring evaluation.

### Statistical analysis

Statistical analysis utilised SPSS version 20.0 (IBM SPSS Statistics, IBM Corporation, New York, NY, USA). Statistical differences between categorical variables were evaluated based on Chi-square testing using an asymptotic or an exact *P*-value, whichever appropriate. The 5-year disease-specific survival (DSS) rates were calculated by Kaplan–Meier (KM) estimation. A log-rank test served in evaluation of statistical significance in the KM analysis. The follow-up time in the DSS evaluation was defined as the period between the last treatment day and the last day of follow-up or date of death from the disease. To minimise the bias in follow-up, the maximum follow-up period for the analysis was 5 years.

Multivariate survival analysis used the Cox proportional hazards model. The analysis included clinically relevant variables with a *P*-value less than 0.1. The proportional hazard assumptions were tested with KM curves. A two-sided *P*-value less than 0.05 was considered statistically significant.

## Results

### *Td*-CTLP and its association with patient and tumour characteristics in OPSCC

**Fig. 1:**
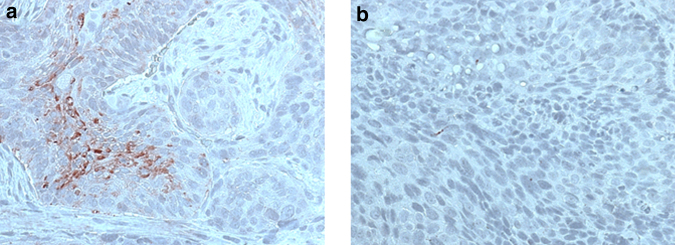
Immunohistochemical staining of oropharyngeal squamous cell carcinoma (OPSCC) samples with *Treponema denticola* chymotrypsin-like protease (*Td*-CTLP) antibody. **a** OPSCC with positive expression of *Td*-CTLP. **b** OPSCC negative for *Td*-CTLP

*Td*-CTLP immunoexpression in the 201 samples occurred as follows: 19% had none (*n* = 39), 31% mild (*n* = 63), 31% moderate (*n* = 63), and 18% strong (*n* = 36) expression. *Td*-CTLP was expressed in the cytoplasm of carcinoma cells in granular and diffuse forms (Fig. 1). It showed a significant association with tumour site of origin, stage, grade of differentiation, presence of regional metastasis (N), and patient smoking habit, but not with primary tumour size (T), gender, or excess alcohol use (Table 1).

**Table 1:** *Treponema denticola* chymotrypsin-like protease (*Td*-CTLP) immunoexpression in relation to patient and tumour characteristics; patient sex, smoking and alcohol consumption, tumour site of origin, grade of differentiation, T class (primary tumour size), N class (presence of regional lymph node metastasis), tumour stage, and human papillomavirus (HPV) status

	Total	None (0)		Mild (1)		Moderate (2)		Strong (3)		*P* -value
		*n*	%	*n*	%	*n*	%	*n*	%	
Sex										0.050
Male	149	32	21	50	34	43	29	24	16	
Female	52	7	13	13	25	20	38	12	23	
Smoking										**0.037**
None	26	4	15	16	62	4	15	2	8	
Finished	49	9	18	17	35	12	24	11	22	
Current	96	16	17	25	26	33	34	22	23	
Excess alcohol consumption										0.421
None	61	11	18	28	46	13	21	9	15	
Finished	24	4	17	3	13	10	42	7	29	
Current	38	8	21	9	24	17	45	4	11	
Tumour site										**0.023**
Anterior wall	61	12	20	17	28	25	41	7	11	
Lateral wall	116	24	21	44	38	28	24	20	17	
Posteroir wall	3	1	33	0	0	0	0	2	67	
Superior wall	21	2	10	2	10	10	48	7	33	
Tumour grade										**0.001**
Grade 1	18	1	6	3	17	8	44	6	33	
Grade 2	78	13	17	23	29	24	31	18	23	
Grade 3	105	25	24	37	35	31	30	12	11	
T class										0.837
T1-2	114	22	19	37	32	35	31	20	18	
T1-3	87	17	20	26	30	28	32	16	18	
N class										**0.001**
N0	39	2	5	7	18	20	51	10	26	
N+	162	37	23	56	35	43	27	26	16	
Tumour stage										**0.007**
I–II	30	2	7	6	20	14	47	8	27	
III–IV	171	37	22	57	33	49	29	28	16	
HPV status										**<0.001**
HPV negative	96	12	13	22	23	36	38	26	27	
HPV positive	105	27	26	41	39	27	26	10	10	

Statistically significant *P*-values (< 0,05) are in bold

More than half of patients (58%) had a tumour arising from the lateral wall of the oropharynx, followed in prevalence by the anterior (30%), superior (10%), and posterior wall (1%). In anterior and lateral-wall carcinomas, none to moderate immunoexpression (score, 0–2) of *Td*-CTLP dominated, whereas in posterior and superior wall carcinomas the major proportion of the tumours had strong *Td*-CTLP immunoexpression (score, 3). *Td*-CTLP staining was more prominent in Grade-1 tumours than in Grade-3 tumours. High *Td*-CTLP immunopositivity was associated with class-N0 disease. Stage I–II disease had higher *Td*-CTLP immunoexpression than did Stage III–IV disease. Expression of *Td*-CTLP was higher among smokers and ex-smokers than among non-smokers (Table 1).

### *Td*-CTLP and its association with treatment of OPSCC

No difference in the *Td*-CTLP immunoexpression existed between patients receiving primary surgery and RT/CRT as adjuvant treatment (*n* = 130) and those treated with definitive RT/CRT (*n* = 71). Furthermore, no difference in the 5-year DSS was observed between the treatment groups.

### *Td*-CTLP and its association with HPV status

**Fig. 2:**
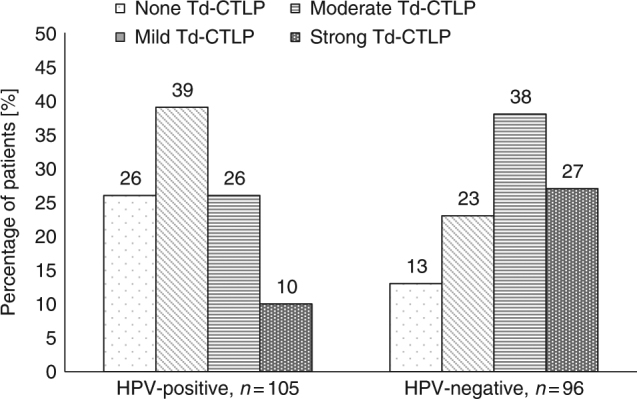
The percentages of *Treponema denticola* chymotrypsin-like protease (*Td*-CTLP) immunoscore values among 201 oropharyngeal squamous cell carcinoma (OPSCC) patients in human papillomavirus (HPV)-positive and HPV-negative subgroups

*Td*-CTLP immunoexpression showed a significant association with HPV status. Strong expression was associated with HPV negativity and mild expression with HPV positivity (Table 1 and Fig. 2).

### *Td*-CTLP and its association with TLR 5, 7, and 9

The association of TLR 5, 7, or 9 and *Td*-CTLP were calculated separately for the HPV-positive and HPV-negative subgroups (Table 2). In the HPV-positive subgroup, among tumours with low TLR 5 expression, *Td*-CTLP expression was low. Consequently, in the HPV-negative subgroup, among tumours with strong expression of TLR 5, *Td*-CTLP expression was high. In the HPV-positive subgroup, high immunoexpression of TLR 7 was associated with lower *Td*-CTLP immunoexpression, whereas in the HPV-negative subgroup, mild immunoexpression of TLR 7 was associated with higher *Td*-CTLP expression. No association existed between TLR 9 and *Td*-CTLP expression.

**Table 2:** *Treponema denticola* chymotrypsin-like protease (*Td*-CTLP) immunoexpression in relation to toll-like receptor (TLR) 5, 7, and 9 expression of 201 oropharyngeal squamous cell carcinoma (OPSCC) patients grouped according to human papillomavirus (HPV) status

	Total	None (0)		Mild (1)		Moderate (2)		Strong (3)		*P* -value
		*n*	%	*n*	%	*n*	%	*n*	%	
HPV-positive OPSCC										
TLR 5										**0.013**
0	47	13	28	24	51	8	17	2	4	
1	27	8	30	7	26	9	33	3	11	
2	23	5	22	7	30	8	35	3	13	
3	8	1	13	3	38	2	25	2	25	
HPV-negative OPSCC										
TLR 5										**0.008**
0	12	1	8	6	50	3	25	2	17	
1	10	2	20	3	30	3	30	2	20	
2	52	9	17	10	19	21	40	12	23	
3	22	0	0	3	14	9	41	10	45	
HPV-positive OPSCC										
TLR 7										**0.013**
1	6	1	17	1	17	4	67	0	0	
2	44	7	16	19	43	10	23	8	18	
3	55	19	35	21	38	13	24	2	4	
HPV-negative OPSCC										
TLR 7										**0.006**
1	40	3	8	7	18	16	40	14	35	
2	42	6	14	8	19	17	40	11	26	
3	14	3	21	7	50	3	21	1	7	
HPV-positive OPSCC										
TLR 9										0.715
0	7	3	43	3	43	1	14	0	0	
1	23	6	26	9	39	7	30	1	4	
2	53	7	13	22	42	12	23	9	17	
3	22	8	36	7	32	7	32	0	0	
HPV-negative OPSCC										
TLR 9										0.291
0	2	1	50	0	0	1	50	0	0	
1	6	2	33	2	33	1	17	1	17	
2	39	2	5.1	9	23	18	46	10	26	
3	49	7	14	11	22	16	33	15	31	

Statistically significant P-values (< 0,05) are in bold

### *Td*-CTLP and its association with survival

**Fig. 3:**
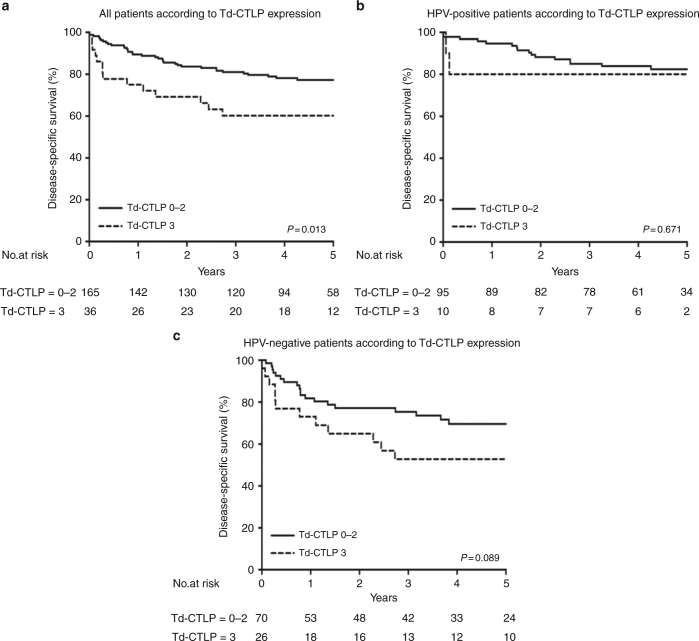
The 5-year disease-specific survival (DSS) rates calculated by Kaplan–Meier (KM) estimation for the entire sample (**a**) and for the human papillomavirus (HPV)-positive (**b**) and the HPV-negative (**c**) subgroups. Samples are grouped according to *Treponema denticola* chymotrypsin-like protease (*Td*-CTLP) immunoexpression to categories none to moderate (score = 0–2) and strong (score = 3)

In survival analysis, no difference in the 5-year DSS was observed when the patient groups stained with none to mild (0–1) and moderate to strong (2–3) *Td*-CTLP were compared. However, patients with *Td*-CTLP score 0–2 had a significantly better 5-year DSS (79%) than did patients with strong *Td*-CTLP immunoexpression (61%) (*P* = 0.013) (Fig. 3). In HPV-positive patients, *Td*-CTLP had no association with DSS (*P* = 0.671). Among patients with HPV-negative OPSCC, the 5-year DSS was 73% among patients with *Td*-CTLP score 0–2 and was 54% among those with strong *Td*-CTLP expression, but this difference was not significant (*P* = 0.089). In multivariate analysis, *Td*-CTLP was never an independent prognostic factor (*P* = 0.115).

## Discussion

We found *Td*-CTLP in the majority (87%) of all HPV-negative OPSCCs. According to the literature, the same common risk factors: smoking and poor oral hygiene, are associated with development of HPV-negative OPSCC^36^ and periodontitis.^37–39^ Our results are in line with these findings, as we found an association between regular smoking and high *Td*-CTLP immunoexpression. In addition, we detected *Td*-CTLP immunopositivity also among 74% of HPV-positive OPSCC samples. Its expression levels were, however, significantly lower than for the HPV-negative subgroup. Interestingly, some studies suggest that chronic periodontitis is a risk factor for HPV-positive base-of-tongue and head-and-neck cancers,^40,41^ and findings support the hypothesis of the periodontal pocket as a reservoir for latent HPV.^42^ Our investigation provides no information as to whether HPV infection is followed by *Td* infection or vice versa in the subgroup of HPV-positive and *Td*-CTLP-positive cancers. Thus, the role of *Td* in HPV-positive OPSCC remains open and requires further examination.

Low *Td*-CTLP immunoexpression was associated with tumours arising from the anterior and the lateral wall of the oropharynx. In addition, low *Td*-CTLP immunopositivity was more prominent in poorly differentiated tumours. These findings are consistent with Goldberg et al.,^43^ who showed HPV-positive tumours as being more likely to have higher differentiation grade and to arise predominantly from the palatine or lingual tonsils. The present finding of an association between lymph node metastasis and low *Td*-CTLP immunoexpression is in line with the results linking HPV-positive OPSCC and higher N class.^43,44^ Further, we detected an association between lower *Td*-CTLP immunoexpression and stage III–IV tumours, which probably is explained by the fact that HPV-positive OPSCC is typically diagnosed at an advanced stage.^5^

TLRs and their role in carcinogenesis have been widely studied. TLRs participate in innate immune response cascades through recognition of pathogen-associated molecular patterns of bacteria, viruses, fungi, and parasites, and non-infectious structures, for example from dead or dying cells associated with tissue damage. That low *Td*-CTLP immunoexpression in the HPV-positive subgroup was associated with low TLR 5 expression and high TLR 7 expression is in line with our earlier results. Those showed HPV-related OPSCC having typically low TLR 5 and high TLR 7 immunoexpression. In addition, earlier we proposed that a high TLR 5 and low TLR 7 expression in HPV-positive OPSCC is associated with poor DSS.^34^ Unfortunately, in the present study, the number of HPV-positive, strong *Td*-CTLP immunoscore samples with high TLR 5 and/or low TLR 7 expression was too small (*n* = 2) for survival evaluation. Nevertheless, our current finding of an association of strong *Td*-CTLP immunoexpression with high TLR 5 expression and low TLR 7 expression in the HPV-negative subgroup suggests that differences exist in the immunological responses between HPV-positive and HPV-negative OPSCC subgroups with *Td* infection, and that these may be linked to the presence of *Td*.

We showed that patients with weaker *Td*-CTLP immunoexpression had significantly better 5-year DSS (79%) than did patients with strong *Td*-CTLP expression (61%). A similar trend, although not statistically significant, was apparent for DSS in HPV-negative OPSCC. The number of patients with strong *Td*-CTLP expression in HPV-positive OPSCC was too small for us to estimate the effect of strong *Td*-CTLP on survival in this subgroup. These findings, however, evoke further questions on the role of *Td* infection on survival of patients with HPV-positive as well as HPV-negative OPSCC, and need to be studied in larger patient series.

## Conclusions

*Td*-CTLP was highly expressed in OPSCC, and was associated with the HPV status of the tumour tissue. High immunoexpression of *Td*-CTLP in HPV-negative OPSCC may be a result of common risk factors for HPV-negative OPSCC and *Td* infection and periodontitis. The role of *Td*-CTLP in immunological responses, carcinogenesis, and clinical outcome needs further investigation both in HPV-negative and HPV-positive OPSCC.
